# No Effect of *Wolbachia* on Resistance to Intracellular Infection by Pathogenic Bacteria in *Drosophila melanogaster*

**DOI:** 10.1371/journal.pone.0040500

**Published:** 2012-07-11

**Authors:** Susan M. Rottschaefer, Brian P. Lazzaro

**Affiliations:** Department of Entomology, Cornell University, Ithaca, New York, United States of America; Centro de Pesquisas René Rachou, Brazil

## Abstract

Multiple studies have shown that infection with the endosymbiotic bacterium *Wolbachia pipientis* confers *Drosophila melanogaster* and other insects with resistance to infection by RNA viruses. Studies investigating whether *Wolbachia* infection induces the immune system or confers protection against secondary bacterial infection have not shown any effect. These studies, however, have emphasized resistance against extracellular pathogens. Since *Wolbachia* lives inside the host cell, we hypothesized that *Wolbachia* might confer resistance to pathogens that establish infection by invading host cells. We therefore tested whether *Wolbachia*-infected *D. melanogaster* are protected against infection by the intracellular pathogenic bacteria *Listeria monocytogenes* and *Salmonella typhimurium,* as well as the extracellular pathogenic bacterium *Providencia rettgeri*. We evaluated the ability of flies infected with *Wolbachia* to suppress secondary infection by pathogenic bacteria relative to genetically matched controls that had been cured of *Wolbachia* by treatment with tetracycline. We found no evidence that *Wolbachia* alters host ability to suppress proliferation of any of the three pathogenic bacteria. Our results indicate that *Wolbachia-*induced antiviral protection does not result from a generalized response to intracellular pathogens.

## Introduction

*Wolbachia* is a genus of maternally inherited, obligate intracellular bacteria that infect a wide range of arthropods and filarial nematodes. It has been estimated that as many as 70% of all insect species may be infected [1]. Extensive horizontal transfer is credited with introducing *Wolbachia* to such a large number of host species. Once introduced, the successful spread of *Wolbachia* throughout host populations can be explained in large part by the ability to act as reproductive parasites, manipulating or disrupting the host reproductive biology in such a way to promote their own transmission. In many species, *Wolbachia* induces cytoplasmic incompatibility (CI), which causes high egg mortality in crosses between infected males and uninfected females, resulting in a relative fitness advantage for infected females and driving *Wolbachia* spread once *Wolbachia* infection has reached a critical threshold in the population [2]. Natural selection could help *Wolbachia* reach that threshold and facilitate further spread if the bacterium provides an additional selective advantage to infected hosts.

In one example of such an advantage, *Drosophila melanogaster* infected with *Wolbachia pipientis* show dramatic resistance to infection by RNA viruses [3], [4]. This antiviral protection appears robust in *D. melanogaster,* having been observed across multiple host genotypes and *Wolbachia* strains [3], [4]. Similar antiviral protection is observed when *D. simulans* is infected with certain *Wolbachia* strains, although other *Wolbachia* strains infecting *D. simulans* do not alter resistance [5]. These observations indicate that *Wolbachia* infection can influence host immunity, but the mechanism of pathogen resistance remains unknown. Previous work in *Drosophila* suggesting that *Wolbachia* infection does not confer protection against secondary bacterial infection has focused on extracellular bacterial pathogens [6]. Like viruses, however, some pathogenic bacteria establish infection by invading host cells where *Wolbachia* is resident. To date, there have been no published tests of whether *Wolbachia* can confer resistance to intracellular bacterial infection.

It has been hypothesized that *Wolbachia* alters the systemic immune response of the host, increasing the ability to quickly detect and mount a response to the infection. In *Aedes aegypti*, for example, *Wolbachia*-induced resistance to a range of pathogens including filarial nematodes, Gram-negative bacteria, and Dengue virus is associated with increased basal expression of immune genes [7], [8], [9]. Microarray analysis of Drosophila S2 cells showed slight upregulation of some genes involved in the Toll and IMD pathways in the presence of *Wolbachia* infection [10], although other studies of selected immune genes in whole flies have found that *Wolbachia* does not alter expression in *D. melanogaster* [6] or *D. simulans* [11]. If *Wolbachia* is able to alter the systemic immune response of *D. melanogaster*, we would expect to see increased resistance against bacterial pathogens in addition to viruses. *Wolbachia* infection does not confer *D*. *melanogaster* or *D. simulans* with resistance against the pathogenic bacteria *Pseudomonas aeruginosa*, *Serratia marcescens,* or *Erwinia carotovora* [6] which are all extracellular pathogens. We hypothesized that *Wolbachia* infection might increase resistance specifically to intracellular pathogens. Intracellular pathogen surveillance could be heightened as a consequence of *Wolbachia* infection, allowing for rapid detection and elimination of pathogens invading the cytoplasm. Alternatively, since *Wolbachia* resides within host cells, it could limit the success of an intracellular pathogen through competition for resources within the host cytoplasm. In either of these cases, increased resistance would only be observed when *Wolbachia-*infected individuals are challenged with an intracellular pathogen.

We investigated whether *Wolbachia* infection alters *D. melanogaster* defense against secondary bacterial infection, and in particular against pathogenic intracellular bacteria. We specifically focused in this paper on resistance, defined as the ability to minimize pathogen burden [12]. We compared the ability to suppress secondary pathogen infection of flies from five isofemale lines of *D. melanogaster* that are naturally infected with *Wolbachia* to the ability of those same lines to suppress pathogenic infection after removal of the *Wolbachia* with tetracycline. To control for the effect of the tetracycline, we also evaluated tetracycline treatment in five naturally *Wolbachia*-uninfected isofemale lines. In order to determine whether *Wolbachia* infection influences generalized resistance to multiple pathogens or a more specific response to intracellular pathogens, we tested infection with *Salmonella typhimurium* and *Listeria monocytogenes*, which are intracellular bacterial pathogens, and *Providencia rettgeri*, an extracellular pathogen. We found no evidence that *Wolbachia* alters resistance to any of the three bacterial pathogens tested.

## Methods

### Flies and Antibiotic Treatment

The *D. melanogaster* isofemale lines used in this experiment were established from field-inseminated females collected in Newfield, New York, USA, in 2005. Each individual female was placed in media-containing vials immediately after collection, and her resulting progeny were allowed to sib-mate. These isofemale lines have been maintained since then by recurrent mass sib-mating. Genetic variation observed among the isofemale lines therefore reflects variation in the natural population from which they were sampled. A diagnostic PCR which amplified *wsp* was used to determine *Wolbachia* infection status of the lines [13]. Antiviral protection has been observed in *D. melanogaster* infected with the *Wolbachia* strains *w*Mel, *w*MelCS and *w*MelPop [3], [4]. There is evidence that *w*Mel is the predominant variant infecting field populations [14], so it is likely to be the strain present in our recently founded lines, although we did not explicitly test this. We randomly chose 5 infected [WOLB(+)] and 5 uninfected [WOLB(–)] lines to use for the experiment. *D. melanogaster* can be experimentally cured of *Wolbachia* by treatment with the antibiotic tetracycline [15]. Flies from both naturally infected and naturally uninfected lines were treated with tetracycline as described below, resulting in four treatment groups to be contrasted for resistance to pathogenic bacterial infection: WOLB(+)TET(–), WOLB(+)TET(+), WOLB(–)TET(–), and WOLB(–)TET(+).

Flies were reared on the standard Cornell Drosophila medium (8.3% w/v glucose, 8.3% w/v brewer’s yeast, 1% w/v agar) throughout the experiment. For the antibiotic treatment, the flies were reared for three generations on the standard Cornell medium with 50 ug/ml tetracycline added [4]. After each generation on tetracycline supplemented medium, eight flies from each line were screened for the presence of *Wolbachia* using the PCR assay described above [13]. Approximately 50% of the flies screened after one generation of tetracycline treatment were cured of *Wolbachia* and approximately 90% were cured after two generations of treatment. After three generations of tetracycline treatment, *Wolbachia* was not detected in any of the flies tested and the isofemale lines were then returned to the standard medium without antibiotic for all subsequent generations. Flies in all treatments were maintained at 25°C with 12 h light, 12 h dark. Flies were infected 1–5 hours after “dawn”. All males used for infections were aged 3–5 days.

### Bacterial Strains

*Providencia rettgeri* strain Dmel is a Gram-negative extracellular pathogen isolated from wild caught *D. melanogaster* that causes moderate mortality in the fly [16]. *Salmonella enterica* serotype Typhimurium S5520 (obtained from Dr. Martin Wiedmann, Cornell University) is a Gram-negative bacterium which is able to establish an intracellular infection causing mortality in *D. melanogaster*, although the bacteria do not replicate to high numbers [17]. *Listeria monocytogenes* 10403S (obtained from Dr. Martin Wiedmann, Cornell University) is a Gram-positive intracellular bacterium which is able to invade and replicate to high numbers within the cells of *D. melanogaster*, causing moderate mortality [18].

### Infections

Since residual effects of tetracycline may persist multiple generations after treatment [19], we measured systemic bacterial load 2, 4, and 6 generations after ending tetracycline treatment. Three sets of infections were done in a day (one for each pathogen) and were repeated on three replicate days for each generation tested. For the infections, 15 males from each line and treatment were anesthetized on CO_2_ and pricked in the thorax with a 0.1 mm pin dipped into a bacterial culture. *P. rettgeri* cultures were grown in LB at 37°C with shaking overnight and diluted to A_600_ = 1 immediately before infections. *L. monocytogenes* cultures were grown in BHI liquid overnight at 37°C with shaking. To prepare the inocula, 2 ml of liquid culture with A_600_ = 1 was spun down and the supernatant removed, and the pellet was resuspended in 200 µl of BHI. *S. typhimurium* cultures were grown in BHI liquid overnight at 37°C without shaking. To prepare the inocula, 2 ml of liquid culture with A_600_ = 1 was spun down and the supernatant removed, and the pellet was resuspended in 200 µl of BHI.

To measure systemic bacterial load, 3 pools of 5 flies from each line were homogenized and plated approximately 24 hours after infection. Flies infected with *P. rettgeri* were homogenized in 500 µl LB, and the homogenate was diluted 1∶100 prior to plating on LB plates. Flies infected with *L. monocytogenes* and *S. typhimurium* were homogenized in 250 µl BHI. The *L. monocytogenes* homogenate was diluted 1∶10 in BHI prior to plating on BHI plates, and the *S. typhimurium* homogenate was not diluted prior to plating on BHI plates. A spiral plater (Don Whitley Scientific) was used to plate 50 µl of each sample over a continuous exponential dilution. Plates were grown at 37°C overnight. The bacteria used for experimental infections grow into visible colonies during this period, while gut commensal bacteria do not appear as visible colonies on the plates until approximately 24 hours later. Thus, we can be certain that the colonies we count reflect systemic pathogen load. Every plate was visually inspected to verify that the color and morphology of all colonies were consistent with that of the experimental bacteria, and any plates with contaminating colonies were discarded. The resulting colonies were counted using the ProtoCOL plate counter associated with the spiral plater to determine the systemic pathogen load of the flies.

### Statistical Analysis

To assess the effect of *Wolbachia* infection, tetracycline treatment, and time since tetracycline treatment on resistance to each pathogen, we performed a mixed-model analyses of variance (ANOVA) on the natural log transformed bacterial load data using the following model:
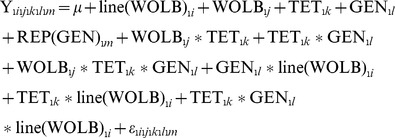
where Y is the natural log of the bacterial load, line(WOLB) (*i* = 1,5) represents the effect of *Drosophila* genetic line within each level of the model factor WOLB, WOLB (*j = 1,2)* represents the *Wolbachia* infection status of each line prior to antibiotic treatment, TET (*k* = 1,2) represents whether or not flies were treated with tetracycline, GEN (*l* = 1,3) represents whether the experiment was performed 2,4, or 6 generations after tetracycline treatment, and REP(GEN) (*m* = 1,3) is the random effect of the replicate day on which the data were collected within each generation. The factor WOLB_j_*TET*_k_* tests for differential effects of tetracycline treatment on *Wolbachia*-infected and *Wolbachia*-uninfected lines, which allows us to distinguish the effect of removing *Wolbachia* from the overall effect of tetracycline. The factor WOLB_j_*TET_k_*GEN*_l_* tests whether effects of tetracycline on *Wolbachia*-infected and uninfected lines are consistent across the successive generations. The factor GEN_l_*line(WOLB)*_i_* tests whether the lines within each WOLB level behave consistently across the generations. The factor TET_k_*line(WOLB)*_i_* tests whether the effect of tetracycline treatment varies among lines within each WOLB level. The factor TET_k_*GEN_l_*line(WOLB)*_i_* tests whether tetracycline treatment has genotype-dependent effects that vary across generations.

**Table 1:** Description of Factors Tested in Analyses of Variance.

Factor	Effect Type	Effect Measured
line(WOLB)	fixed	effect of each genetic line nested within the factor WOLB
WOLB	fixed	*Wolbachia* status of each line prior to tetracycline treatment
TET	fixed	whether or not flies were treated with tetracycline
GEN	fixed	number of generations since tetracycline treatment (2, 4, or 6)
REP(GEN)	random	replicate day on which the experiment was performed
WOLB*TET	fixed	differential effects of tetracycline on flies with and without *Wolbachia*
TET*GEN	fixed	differential effects of tetracycline across the generations tested
GEN*line(WOLB)	fixed	differential effects of line across the generations tested
TET*line(WOLB)	fixed	differential effects of tetracycline on flies of each line
WOLB*TET*GEN	fixed	different effects of tetracycline on flies with and without *Wolbachia* and across generations
TET*GEN*line(WOLB)	fixed	differential effects of tetracycline on flies of each line and across generations

To further elucidate the nature of the observed effect of the line(WOLB)_i_*TET*_k_* *GEN*_l_* interaction on resistance to *P. rettgeri* (see Results), we performed an additional mixed ANOVA for each generation separately. This model takes the form:
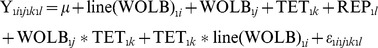
where Y is the natural log of the bacterial load, line(WOLB) (*i* = 1,5) represents the effect of genotype nested within each level of the factor WOLB, WOLB (*j = 1,2)* represents the *Wolbachia* infection status of each line prior to antibiotic treatment, TET (*j* = 1,2) represents whether or not flies were treated with tetracycline, and REP (*k* = 1,3) is the random effect of the replicate day on which the experiment was performed. The factor WOLB_j_*TET*_k_* tests for differential effects of tetracycline treatment on *Wolbachia*-infected and *Wolbachia*-uninfected lines. The factor TET_j_*line(WOLB)*_i_* tests whether tetracycline treatment has genotype-dependent effects.

All of the model factors described in the text above are also listed in Table 1. Removal of various non-significant factors from the model does not change the qualitative outcome of any of our analyses, so we present here the full models in order to provide the most complete information. All analyses were performed using SAS 9.3 (SAS Institute).

**Figure 1:**
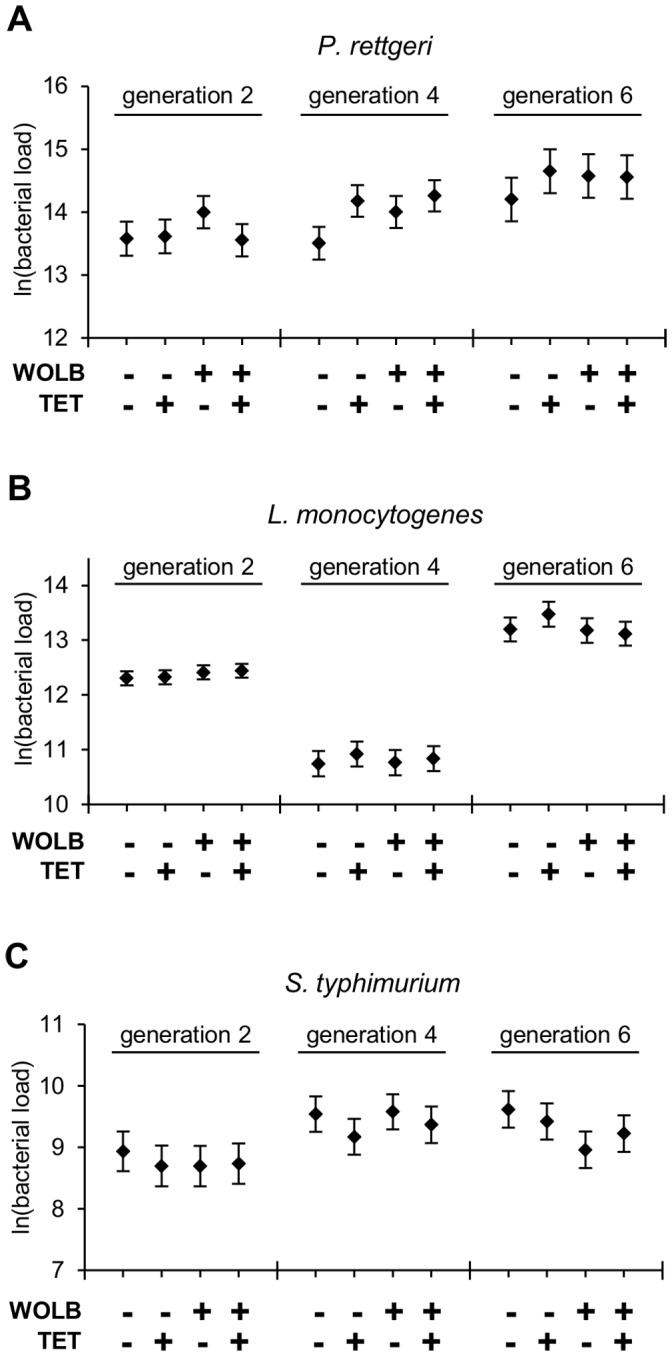
Systemic bacterial load is not influenced by *Wolbachia* infection. Least squares means for bacterial load (±1SE) of five *Wolbachia*-infected lines [WOLB(+)TET(-)] and genetically matched lines that have been cured of *Wolbachia* [WOLB(+)TET(+)], as well as five *Wolbachia*-uninfected lines [WOLB(-)TET(-)] and genetically paired tetracycline treated lines[WOLB(-)TET(+)]. Note that the WOLB category on the x-axis refers to initial *Wolbachia*-infection status prior to antibiotic treatment, rather than infection status at the time of experimental infections. Bacterial load was measured 24 hours after infection with the pathogenic bacteria (A) *P. rettgeri* (B) *L. monocytogenes* and (C) *S. typhimurium*. Assays were performed 2, 4, and 6 generations after ending tetracycline treatment, with three replicates in each generation. For each replicate, bacterial load was measured in 3 pools of 5 flies from every line.

**Table 2:** Analyses of variance for fixed effects relating genotype, *Wolbachia* status, tetracycline treatment, and generation to bacterial load.

		*P. rettgeri*		*L. monocytogenes*		*S. typhimurium*	
Factor	d.f.	F-ratio	P-value	F-ratio	P-value	F-ratio	P-value
line(WOLB)	8	21.10	<0.0001	14.01	<0.0001	2.26	0.0222
WOLB	1	2.94	0.0873	1.13	0.2880	1.07	0.3020
TET	1	1.60	0.2062	1.03	0.3117	0.79	0.3740
GEN	2	3.93	0.0811	0.19	0.8324	0.02	0.9800
WOLB*TET	1	2.96	0.0860	0.12	0.7254	1.30	0.2548
WOLB*TET*GEN	2	0.01	0.9892	0.48	0.6206	0.09	0.9099
TET*GEN	2	2.11	0.1219	0.15	0.8623	0.56	0.5705
GEN* line(WOLB)	16	1.37	0.1543	0.71	0.7841	0.49	0.9514
TET* line(WOLB)	8	1.88	0.0609	1.42	0.1857	0.83	0.5767
TET*GEN*line(WOLB)	16	2.01	0.0117	1.30	0.1910	1.51	0.0910

**Table 3:** Analyses of variance relating fixed effects of genotype, *Wolbachia* status, and tetracycline treatment to bacterial load when infected with *P. rettgeri* 2, 4, and 6 generations after tetracycline treatment.

		generation 2		generation 4		generation 6	
Factor	d.f.	F-ratio	P-value	F-ratio	P-value	F-ratio	P-value
line(WOLB)	8	8.33	<0.0001	6.69	<0.0001	9.24	<0.0001
WOLB	1	1.02	0.3146	1.64	0.2029	0.39	0.5311
TET	1	0.68	0.4117	3.91	0.0497	1.12	0.2918
TET*WOLB	1	1.08	0.2999	0.83	0.3646	1.01	0.3176
TET* line(WOLB)	8	3.30	0.0017	0.61	0.7707	1.91	0.0619

## Results

When flies were infected with *P. rettgeri,* we observed significant differences in bacterial load across the isofemale lines (*p*<0.0001, Table 2), but no difference in bacterial load owing to the initial *Wolbachia* status of those lines (*p*  = 0.0873). Systemic pathogen load of tetracycline-treated flies, considered across genotypes, did not differ from that of untreated flies (*p*  = 0.2062). The effect of tetracycline treatment on *Wolbachia*-infected lines was not different from the effect of tetracycline treatment on *Wolbachia-*uninfected lines (WOLB*TET, *p*  = 0.0860, Table 2 and Figure 1A), indicating that removal of *Wolbachia* does not influence ability to suppress *P. rettgeri* infection. Interestingly, we find a nearly significant TET*line(WOLB) interaction (*p  = *0.0610, Table 2), which suggests that the effects of tetracycline may be stronger in some genetic backgrounds than others. Additionally, the three-way TET*GEN* line(WOLB) interaction is significant (*p*  = 0.0117, Table 2). This three way interaction indicates that the genotype-specific effect of tetracycline treatment varies across generations, but it does not provide any direct information about the nature of this complex interaction. We decided to investigate this three-way interaction further by running a separate analysis for each of the three generations tested. Interestingly, we find a significant TET*line(WOLB) interaction in response to *P. rettgeri* two generations after treatment (*p  = *0.0017, Table 3) whereas this interaction is not significant in the subsequent generations. Taken together, these results suggest that an effect of tetracycline may persist in some, but not other genetic backgrounds two generations after treatment, but that the effect does not persist for four or more generations in any of the genetic backgrounds.

When flies were infected with *L. monocytogenes,* we observed significant differences in bacterial load across the isofemale lines (*p*<0.0001, Table 2), but no difference in *L. monocytogenes* load owing to the initial *Wolbachia* status of those lines (*p*  = 0.2880) or to tetracycline treatment (*p*  = 0.3117). The effect of tetracycline treatment on *Wolbachia*-infected lines was not different from the effect of tetracycline treatment on *Wolbachia-*uninfected lines (WOLB*TET, *p*  = 0.7254, Table 2 and Figure 1B), indicating that removal of *Wolbachia* does not influence ability to suppress *L. monocytogenes* infection. In contrast to infection with *P. rettgeri*, there was no genotype-by-treatment interaction in response to *L. monocytogenes* infection (*p*  = 0.1857), nor was there any indication of a three way genotype-by-treatment-by-generation interaction (*p  = *0.1910).

When flies were infected with *S. typhimurium,* we observed significant differences in bacterial load across the isofemale lines (*p*  = 0.0222, Table 2), but no effect of initial *Wolbachia* status (*p*  = 0.3020) or tetracycline treatment (*p*  = 0.3740). The effect of tetracycline treatment on *Wolbachia*-infected lines was not different from the effect of tetracycline treatment on *Wolbachia-*uninfected lines (WOLB*TET, *p*  = 0.2548, Table 2 and Figure 1C), indicating that removal of *Wolbachia* does not influence ability to suppress infection by *S. typhimurium.* As with infection by *L. monocytogenes*, there was no genotype-by-treatment interaction in response to *S. typhimurium* infection (*p*  = 0.5767) and no three way genotype-by-treatment-by-generation interaction (*p  = *0.0910).

## Discussion

In this experiment we used two intracellular bacterial pathogens and one extracellular bacterial pathogen to investigate whether *Wolbachia* infection influences *D. melanogaster* resistance to pathogenic bacteria. Unfortunately there are no known natural intracellular bacterial pathogens of *D. melanogaster*, so for this experiment we used the human pathogens *Listeria monocytogenes* and *Salmonella typhimurium.* We did not find evidence that *Wolbachia* confers protection against either of the intracellular bacteria. Although these are not natural pathogens of *D. melanogaster*, both are able to invade and replicate within the cells of *D. melanogaster* and have been used to study intracellular infection in *D. melanogaster* [17], [18]. When *Wolbachia*-infected *D. melanogaster* are infected with DCV or Nora virus, both of which are natural pathogens, survival is increased and viral proliferation is inhibited [4]. Increased survival is also observed in *Wolbachia-*infected flies infected with the non-natural pathogen FHV, but in this case viral proliferation does not appear to be inhibited [4]. This observed disconnection between virus proliferation and host mortality suggests that the mechanisms by which *Wolbachia* confers protection involve both host immunity and host tolerance, the effects of which may be specific to particular pathogens or natural host-pathogen pairs. In future studies it may be interesting to test whether similar infection phenotypes are observed with natural intracellular bacterial pathogens.

Likewise, it would also be of interest to investigate the effects of *Wolbachia* on host fitness over the course of an infection. In this experiment we measured resistance, defined as the ability to minimize pathogen burden [12], because we were specifically interested in whether the presence of *Wolbachia* influences the host ability to suppress secondary bacterial infection. *Wolbachia* infection could conceivably also increase host tolerance of infection, such that *Wolbachia*-infected flies might survive longer or have higher reproductive success than uninfected flies despite similar pathogen infection loads. However, *Wolbachia* infection has previously been reported to have no effect on mortality in *D. melanogaster* after infection with extracellular bacterial pathogens [6].

In addition to investigating the effect of *Wolbachia* on resistance to bacterial pathogens, we examined the residual effect of tetracycline on flies multiple generations after treatment. Reduced mitochondrial metabolism and increased mtDNA density have been reported in *D. simulans* two generations after treatment with tetracycline [19], and antibiotic treatment additionally eliminates commensal gut microbes. Gut microbes have important regulatory effects on the immune system in the gut, and the presence or absence of individual microbes can disrupt gut homeostasis [20]. For example, aseptically reared *Anopheles gambiae* are more susceptible to *Plasmodium falciparum* infection than are non-sterile mosquitoes [21]. Two generations after cessation of tetracycline treatment, we found a significant line-by-tetracycline interaction on the ability of flies to suppress infection by the Gram-negative extracellular pathogen *P. rettgeri.* This suggests that residual effects of tetracycline may persist in some, but not other, genetic backgrounds for multiple generations. We speculate that there may be genetic variation for the number of generations required to recover from the effects of tetracycline treatment, perhaps resulting from differences in the ability to reacquire commensal gut microbes and return gut homeostasis or to differences in the rate of mitochondrial recovery. Additional research is required to elucidate the nature of this interaction.

In summary, it is well established that *Wolbachia* provides protection against RNA viruses in *Drosophila* [3], [4] so we sought to determine whether the *Wolbachia*-induced resistance to viruses could be generalized to other intracellular pathogens. We measured the abilities of *Wolbachia*-infected and uninfected *D. melanogaster* to suppress infection by the intracellular pathogenic bacteria *L. monocytogenes* and *S. typhimurium* and the extracellular pathogenic bacterium *P. rettgeri*, but we observed no effect of *Wolbachia* on resistance to infection by any of the three, irrespective of how they colonize the host.
